# Multivariable analysis of total cholesterol levels in male Swiss Armed Forces conscripts 2006-2012 (*N* = 174,872)

**DOI:** 10.1186/s12872-016-0218-2

**Published:** 2016-02-17

**Authors:** Marcel Bruggisser, Dieter Burki, Martin Haeusler, Frank J. Rühli, Kaspar Staub

**Affiliations:** Swiss Armed Forces, Medical Service, Worblentalstrasse 36, Ittigen, CH-3063 Switzerland; Viollier AG, Hagmattstrasse 14, Allschwil, CH-4123 Switzerland; Institute of Evolutionary Medicine, University of Zürich, Winterthurerstrasse 190, CH-8057 Zürich, Switzerland; Institute of Anatomy, University of Zürich, Winterthurerstrasse 190, Zürich, CH-8057 Switzerland

**Keywords:** TCL, Groups at risk, Obesity

## Abstract

**Background:**

Cholesterol is an important contributor to morbidity and mortality risks due to its association with obesity, cardiovascular disease, and cancer. A system of mandatory military conscription is a useful tool for disease-risk monitoring in a given male population. Swiss military conscription data are representative for more than 90 % of a given male birth cohort (with Swiss citizenship). The medical examination also includes voluntary laboratory testing, for which approximately 65 % of the young men present at conscription give consent.

**Methods:**

Here we present the temporal and subgroup analyses of total serum cholesterol levels (TCL) among Swiss conscripts from 2006 to 2012 (N = 174,872; mean age = 19.75 years). The voluntary blood samples were tested by a central laboratory (Viollier AG) with identical measurement standards and strict quality control. To test differences in TCL by socioeconomic occupational status, sports test performance, Body Mass Index (BMI), age, and place of residence of the conscripts we used a multivariable regression model with TCL as dependent variable.

**Results:**

Mean TCL decreased significantly, by 0.125 mmol/l (95 % CI 0.108–0.142, *p* < 0.001) from 4.225 mmol/l (95 % CI 4.210–4.240) in 2006 to 4.100 mmol/l (95 % CI 4.091–4.109) in 2012. Similarly, the prevalence of conscripts with an elevated TCL ≥ 5.17 mmol/l decreased from ≥10.2 % prior to 2011 to 6.9 % in 2011 and 8.2 % in 2012. Multivariate regression showed an association between elevated TCL and lower socioeconomic occupational status, lower sports test performance, higher BMI, higher age, and area of residence. There was no longer a significant increase in mean TCL among the three grades of obesity (BMI ≥ 30.0 kg/m2) as defined by the WHO. Within the BMI categories of normal weight and overweight, TCL was stratified by sports performance (better sports performance = lower TCL).

**Conclusion:**

Decreasing TCL in 2011 and 2012 fits the known pattern of conscripted persons’ stabilizing BMI and sports test performance of the conscripts in recent years. However, small temporal drifts within the laboratory analyses cannot be ruled out as confounding factors. In conclusion, identifying subgroups with unfavorable lipid profiles will contribute to the continuing success of intensified public health programs.

## Background

Elevated serum cholesterol levels are an important contributor to an increase in morbidity and mortality due to their association with obesity, cardiovascular disease, and certain types of cancer [1, 2]. Obesity and abnormal plasma lipid levels are part of the so-called metabolic syndrome [3]. Usually, total cholesterol level (TCL), high-density lipoprotein (HDL) cholesterol, low-density lipoprotein (LDL) cholesterol and triglycerides (TG) together with a risk stratification model are used to determine whether a patient should be recommended a change of life style or be treated with a lipid-lowering medication [4, 5]. Although HDL-C, LDL-C and triglyceride levels are generally all used for targeting and monitoring the therapy [5], they significantly correlate with TCL [6]. Thus, TCL has been shown to perform only slightly inferior than the TC/HDL ratio as a predictor of cardiovascular risk [7]. In fact, for the general male population, the inclusion of HDL-C in risk estimation results in only a modest improvement in overall risk estimation [6, 8]. This suggests that TCL can be used as good initial screening parameter [9]. TCL itself has been identified as a valuable predictor in assessing cardiovascular risk [1]: On the one hand, elevated TCL has been shown to substantially increase cardiovascular disease, including coronary heart disease, and overall mortality in younger men. On the other hand, men with a favorable baseline TCL had an estimated life expectancy 3.8 to 8.7 years longer than that of men with an elevated TCL [1]. Generally, a serum TCL of less than 5.17 mmol/l is desirable, whereas a value between 5.17 and 6.18 mmol/l is classified as “borderline high,” and a value above 6.18 mmol/l as “high” [10].

A cholesterol-level analysis is part of the medical examination performed in several European armed forces [11, 12]. The medical data of Swiss conscripts provide an annual picture of the health status of the almost entire ~19-year-old male population [13]. Despite being limited to those young men with Swiss citizenship, the population-representative nature and consistency of examination standards makes the health status of conscripts a valuable screening tool for public-health research. The medical examination also includes voluntary laboratory testing, for which approximately 65 % of the young men consent at conscription. TCL analysis has been part of the voluntary laboratory test since 2004. Although three studies analyzed TCL from a cross-sectional perspective in conscript census years 2004, 2005, and 2004-2007, respectively [14–16], no study has yet focused on temporal trends, differences by area of residence (*Grossregion*) or by socio-economic occupational status.

Here, we analyze the temporal changes of TCL and its association with the afore-mentioned possible influencing factors. We limit our study to the period of 2006 - 2012 because TCL was then measured with identical laboratory test devices and standards. Switzerland serves as an excellent example of an industrialised country in Central Europe where differences between three major cultural groups (=the language regions or *Grossregionen*) can be analysed based on such numerous and representative data. In times of increasingly restrictive public-spending budgets, defining groups at risk might be useful to determine the population subgroups of young males that will most benefit from health promotion programs aiming to lower cholesterol levels at an early age in order to substantially decrease morbidity and mortality risk later in life.

## Methods

### Swiss conscription

The regulations of the Swiss federal authority specify that all young Swiss men be called up for conscription during the year in which they turn 19. Conscription either slightly earlier or later is possible upon request. The medical assessments that are part of the conscription process include not only the measurement of height and weight but also the recording of the socioeconomic status (indicated by current occupation) and the place of residence of every conscript, including those who subsequently receive either a deferral or an exemption. The medical examinations are made under professional medical supervision at six dedicated conscription centers with identical qualitative standards for technical equipment and organizational structures. Previous studies have shown that Swiss conscription data are representative for more than 90 % of a given male birth cohort [17]. However, the medical causes of the up to 10 % classified in absentia as unfit for service include the full range of severe diseases and severe physical and psychiatric disabilities. Because of the size of this group and the fact that the list of all possible reasons is not limited to diseases linked to lipid profiles, absenteeism was not considered to have an appreciable effect on the cholesterol distribution.

The multiday recruitment concept also includes a sports performance test (*Test Fitness Rekrutierung*, or TFR) consisting of five components: speed-strength of the legs, speed-strength of the arms, muscular strength of the global trunk, coordination and endurance (maximum number of points: 125). Approximately 80 % of the present conscripts are physically evaluated by the sports performance test/TFR [18, 19]. The medical examination of the conscripts includes a voluntary laboratory test, for which approximately 65 % of the young men consent at conscription. The blood samples are taken by medical personnel at the conscription centers (fasting status is not recorded) and shipped to a laboratory center in Allschwil (Viollier AG) to be tested by state-of-the-art equipment and assays, usually within 12 h by laboratory personnel. Until 2013, the laboratory test included a small chemistry profile including TCL (no cholesterol subfractions were determined). TCL was measured by enzymatic assay (‘CHOL_2’) redundantly on two Siemens Advia 1650 (Siemens Healthcare Diagnostics AG, Zürich, Switzerland). In August 2012, two Siemens Advia 1800 analyzers replaced them; however, the new devices are identical to their predecessors in terms of measurement techniques and reagents. The laboratory ensured application of identical measurement standards (evaluated by regular internal and external quality control) during the entire time period under observation.

### Data and study population

Fully anonymized individual conscription records for the period 01/01/2004-31/12/2012 (N = 345,684) were provided by the Swiss Army (Logistikbasis der Armee - Sanität) in February 2013 under contractual agreement with the study authors. The data included date of birth, date of conscription, height (cm), weight (kg), current occupation (recorded as free-text entry), postcode of place of residence, stage of conscription (first, regular visit versus reassessment, NIAX-code), sports test/TFR results (1–125 points), and TCL (mmol/l). To ensure temporal comparability, we restricted our analyses to the time period during which TCL was measured with identical laboratory test devices and standards (11/07/2006-31/12/2012, N = 269,636). In addition, we restricted our dataset to male conscripts appearing for the first, regular assessment in the recruitment centers (NIAX conscription status code = S, N = 265,569). The dataset was checked for implausible height, weight, sports test results and cholesterol level values; none were found. We calculated BMI (weight [kg]/height [m]^2^) and age at conscription based on date of birth and date of conscription. BMI was categorized according to the official WHO subgroups for underweight (BMI < 18.5 kg/m^2^), normal weight (BMI 18.5–24.9 kg/m^2^), overweight (BMI 25.0–29.9 kg/m^2^) and obesity (BMI ≥ 30.0 kg/m^2^, including the official subcategories) [20]. Furthermore, each conscript was assigned to one of seven regions (*Grossregionen*: Région Lémanique, Mittelland, Nordwestschweiz, Zürich, Ostschweiz, Zentralschweiz, Ticino) on the basis of the ZIP-code of his residential community [17]. We also converted free-text entries of the current occupation to the International Socio-Economic Index of occupational status (ISEI) code as described by Ganzeboom et al. [21]. The ISEI major groups (“low,” “medium,” and “high”) were then built by dividing the ISEI scores into tertiles according to Panczak et al. [22]. We created separate categories for individuals who were still in school (“pupils”) and for those with insufficient or missing data for the occupational status (“imprecise”). The individual sports test/TFR scores (1-125 points, available for N = 225,388 or 84.9 %) were then categorized into tertiles, (“low”, “medium”, “high”), in order of increasing physical performance. Further, elevated TCL was categorized in “borderline high” (5.17-6.18 mmol/l) and “high” (≥6.18 mmol/l) [10].

Excluding individuals not participating in the voluntary laboratory test, the sample for which TCL was available consisted of 174,872 conscripts (65.8 %, Appendix Table 4). Overall, voluntary blood-test participants and non-participants were similar in terms of mean age – 19.75 years (95 % CI 19.75–19.76) versus 19.89 years (95 % CI 19.88–19.89) – and mean BMI – 23.39 kg/m^2^ (95 % CI 23.37–23.40) versus 23.40 kg/m^2^ (95 % CI 23.37–23.42). The yearly comparison between participants and non-participants is reported in Appendix Table 4: The blood-analyses participation rate was steady, lying between 60.4 % and 68.5 % for each of the conscription years between 2006 and 2012 (overall 65.8 %). Age distribution was very similar among participants and non-participants, with the 19-year-old conscripts contributing 45.4 % and 43.6 %, respectively, to the whole dataset and both the 18-year-old and the 20-year-old conscripts contributing 20.3 % to 22.7 %. Regarding socioeconomic occupational status (ISEI), the participant and the non-participant populations were comparable as well. However, non-participants were – in a low single-digit percentage – slightly less overweight, more obese and had slightly lower sports test results than the participants. The greatest differences between participants and non-participants were found in their regional origins: Non-participants were more likely to be residents of Région Lémanique and Nordwestschweiz and less likely to be from Zürich and Ostschweiz, whereas the percentages of participants from Mittelland, Zentralschweiz, and Ticino were again comparable to those of non-participants. All of the percentages reported in Appendix Table 4 are surprisingly stable over time when single conscription years are compared. The total number of conscripts in the first analyzed conscription year, 2006 (N = 18,502), is lower than the total number in the other years (N between 39,115 and 43,201) because only laboratory tests after 11/07/2006 were included.

### Statistical analyses

To test the association between TCL and draft year, age, socioeconomic status, BMI, sports test performance, and regional origin, a multivariable linear regression analysis with TCL as dependent variable was performed. We further controlled TCL among BMI subgroups by sports test performance/TFR. Stata version 13 (Stata Corporation, College Station, TX, USA) was used for all analyses.

### Ethics statement

The data and the permission to use them were available from the Swiss Armed Forces (*Logistikbasis der Armee*) upon submission and approval of a study protocol. According to the bilaterally signed data contract, the Swiss Armed Forces fully anonymized the records by removing all names, social security numbers, and exact residential addresses prior to delivery to the study authors. The anthropometric and laboratory data used in this study are considered nonclinical, governmental data. According to Swiss federal law (*Bundesgesetz über die militärischen Informationssysteme MIG*, BG 510.91, Art. 2, 9, 24–29), the Swiss Army is authorized to make these data accessible in an anonymous form for academic research. Furthermore, the conscripts sign a detailed informed consent form for the voluntary laboratory test (available from the Swiss Army upon request). As for anonymized and nonclinical governmental data, no additional ethical approval is needed (Swiss data privacy act, SR 235.1; 19.6.1992 and Federal Act on Research involving Human Beings HRA, 810.30; 1.1.2014).

## Results

**Fig. 1:**
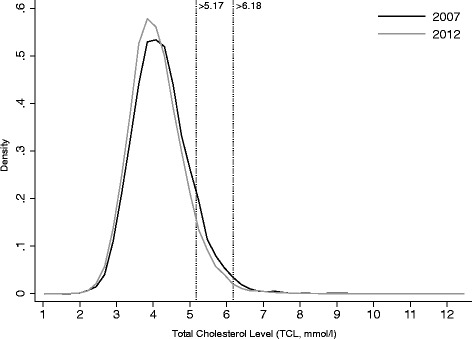
Distribution (density plot) of TCL among male Swiss conscripts 2007 (the first complete conscription year analysed, *N* = 27,258, Mean = 4.191 mmol/l, 10.5 % ≥ 5.17 mmol/l) vs. 2012 (*N* = 26,867, Mean = 4.100 mmol/l, 8.2 % ≥ 5.17 mmol/l)

Mean TCL of the entire study population 2006-2012 (N = 174,872) was 4.175 mmol/l (95 % CI 4.171–4.179, SD = 0.76) with a median of 4.1 mmol/l. The distribution was slightly right-skewed (skewness = 0.715) (Fig. 1). 10.0 % of the young men had an elevated TCL (>5.17 mmol/l), including 8.6 % with a “borderline high” TCL (5.17–6.18 mmol/l), and 1.4 % with a “high” TCL (>6.18 mmol/l).

**Fig. 2:**
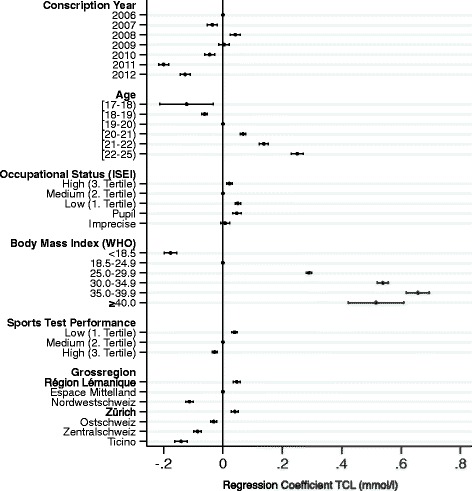
Linear regression coefficient plot TCL among Swiss conscripts 2006-2012 (*N* = 158,836, Table 2)

Over the course of the observation period, average TCL decreased significantly: by 0.125 mmol/l (95 % CI 0.108–0.142, *p* < 0.001), from 4.225 mmol/l (95 % CI 4.210–4.240) in 2006 to 4.100 mmol/l (95 % CI 4.091–4.109) in 2012 (Table 1). Mean TCL among young men conscripted during 2011 and 2012 were particularly lower (<4.190 mmol/l) than mean TCL prior to 2011. Accordingly, the prevalence of “borderline high” TCL (5.17–6.18 mmol/l) decreased from ≥8.7 % prior to 2011 to 5.9 % in 2011 and 7.3 % in 2012. Similar, the prevalence of “high” TCL (>6.18 mmol/l) decreased from ≥1.5 % prior to 2011 to 1.0 % in 2011 and 0.9 % in 2012. Overall, the prevalence of elevated TCL (>5.17 mmol/l) decreased from ≥10.2 % prior to 2011 to 6.9 % in 2011 and 8.2 % in 2012. Linear regression confirmed these results, with the years 2011 and 2012 showing significantly lower values than 2006 (Table 2 and Fig. 2).

**Table 1:** Descriptive statistics of TCL among male Swiss conscripts 2006-2012

		*N*	Mean (95 %-CI)	SD	Median	% [5.17–6.18)	% ≥ 6.18	% ≥ 5.17
Year	2006	11178	4.225 (4.210–4.240)	0.78	4.2	10.0	1.5	11.5
	2007	27258	4.191 (4.182–4.200)	0.77	4.1	9.0	1.5	10.5
	2008	26526	4.278 (4.269–4.287)	0.77	4.2	10.7	1.8	12.5
	2009	27084	4.244 (4.235–4.253)	0.76	4.2	9.8	1.6	11.4
	2010	28402	4.190 (4.181–4.199)	0.76	4.1	8.7	1.5	10.2
	2011	27557	4.033 (4.024–4.042)	0.73	4.0	5.9	1.0	6.9
	2012	26867	4.100 (4.091–4.109)	0.73	4.0	7.3	0.9	8.2
	Total	174872	4.175 (4.171–4.179)	0.76	4.1	8.6	1.4	10.0
Age	[18–19)	39683	4.066 (4.059–4.073)	0.72	4.0	6.3	0.8	7.1
	[19–20)	79354	4.151 (4.146–4.156)	0.75	4.1	8.1	1.2	9.3
	[20–21)	37032	4.242 (4.234–4.250)	0.77	4.2	9.9	1.7	11.6
	[21–22)	11728	4.328 (4.314–4.342)	0.79	4.3	11.6	2.3	13.9
	Total	174872	4.175 (4.171–4.179)	0.76	4.1	8.6	1.4	10.0
SEP (ISEI)	Low	52985	4.207 (4.200–4.214)	0.77	4.1	9.3	1.5	10.8
	Medium	50355	4.155 (4.148–4.162)	0.75	4.1	8.3	1.3	9.6
	High	44320	4.169 (4.162–4.176)	0.75	4.1	8.4	1.3	9.7
	Pupil	14946	4.162 (4.150–4.174)	0.75	4.1	8.0	1.3	9.3
	Imprecise	12266	4.163 (4.149–4.177)	0.78	4.1	8.6	1.6	10.2
	Total	174872	4.175 (4.171–4.179)	0.76	4.1	8.6	1.4	10.0
BMI	<18.5	5561	3.919 (3.902–3.936)	0.67	3.9	3.6	0.4	4.0
	18.5–24.9	126078	4.087 (4.083–4.091)	0.71	4.0	6.4	0.8	7.2
	25.0–29.9	34069	4.405 (4.396–4.414)	0.81	4.3	14.1	2.6	16.7
	30.0–34.9	7042	4.673 (4.652–4.694)	0.89	4.6	22.1	5.4	27.5
	35.0–39.9	1698	4.807 (4.764–4.850)	0.89	4.7	25.4	7.5	32.9
	≥40.0	424	4.692 (4.612–4.772)	0.84	4.6	23.6	4.5	28.1
	Total	174872	4.175 (4.171–4.179)	0.76	4.1	8.6	1.4	10.0
Sports Test	1st Tertile (low)	50268	4.268 (4.261–4.275)	0.82	4.2	11.3	2.2	13.5
	2nd Tertile (medium)	52800	4.143 (4.137–4.149)	0.74	4.1	7.7	1.1	8.8
	3rd Tertile (high)	55768	4.089 (4.083–4.095)	0.70	4.0	6.3	0.7	7.0
	Total	158836	4.164 (4.160–4.168)	0.75	4.1	8.3	1.3	9.6
Region	Région Lémanique	26792	4.268 (4.259–4.277)	0.78	4.2	10.4	1.9	12.3
	Espace Mittelland	44793	4.185 (4.178–4.192)	0.76	4.1	8.6	1.4	10.0
	Nordwestschweiz	17501	4.090 (4.079–4.101)	0.74	4.0	7.1	0.9	8.0
	Zürich	28005	4.243 (4.234–4.252)	0.78	4.2	10.1	1.7	11.8
	Ostschweiz	32610	4.147 (4.139–4.155)	0.75	4.1	8.4	1.2	9.6
	Zentralschweiz	19862	4.097 (4.087–4.107)	0.74	4.0	6.8	1.1	7.9
	Ticino	5309	4.025 (4.006–4.044)	0.71	4.0	5.7	0.6	6.3
	Total	174872	4.175 (4.171–4.179)	0.76	4.1	8.6	1.4	10.0

**Table 2:** Linear regression results of TCL among male Swiss conscripts 2006-2012 (*N* = 158,836)

		Coef.	Std. Err.	t	*p*	−95 % CI	+95 % CI
Year	2006	reference					
	2007	−0.036	0.009	−4.13	0.000	−0.053	−0.019
	2008	0.042	0.009	4.78	0.000	0.025	0.059
	2009	0.004	0.009	0.41	0.680	−0.014	0.021
	2010	−0.045	0.009	−5.20	0.000	−0.062	−0.028
	2011	−0.200	0.009	−23.05	0.000	−0.217	−0.183
	2012	−0.128	0.009	−14.65	0.000	−0.145	−0.110
Age	<18	−0.123	0.046	−2.68	0.007	−0.212	−0.033
	[18–19)	−0.062	0.005	−13.20	0.000	−0.071	−0.053
	[19–20)	reference					
	[20–21)	0.068	0.005	13.97	0.000	0.058	0.077
	[21–22)	0.138	0.008	17.77	0.000	0.123	0.153
	>22	0.250	0.010	24.43	0.000	0.230	0.270
SEP (ISEI)	Low	0.050	0.005	10.63	0.000	0.041	0.060
	Medium	reference					
	High	0.022	0.005	4.30	0.000	0.012	0.032
	Pupil	0.047	0.007	6.51	0.000	0.033	0.061
	Imprecise	0.007	0.008	0.93	0.351	−0.008	0.023
BMI	below 18.5	−0.177	0.011	−16.60	0.000	−0.198	−0.156
	18.5–24.9	reference					
	25.0–29.9	0.290	0.005	61.18	0.000	0.281	0.299
	30.0–34.9	0.538	0.010	55.46	0.000	0.519	0.558
	35.0–39.9	0.657	0.020	33.07	0.000	0.618	0.696
	≥40.0	0.515	0.048	10.75	0.000	0.421	0.609
Sports Test	1st Tertile (low)	0.039	0.005	8.42	0.000	0.030	0.048
	2nd Tertile (medium)	reference					
	3rd Tertile (high)	−0.028	0.004	−6.30	0.000	−0.037	−0.019
Region	Région Lémanique	0.047	0.006	7.62	0.000	0.035	0.059
	Espace Mittelland	reference					
	Nordwestschweiz	−0.113	0.007	−16.81	0.000	−0.126	−0.100
	Zürich	0.040	0.006	6.75	0.000	0.029	0.052
	Ostschweiz	−0.031	0.006	−5.61	0.000	−0.042	−0.020
	Zentralschweiz	−0.086	0.006	−13.48	0.000	−0.098	−0.073
	Ticino	−0.141	0.011	−13.03	0.000	−0.162	−0.120
	constant	4.118	0.009	454.80	0.000	4.100	4.136
N	158836						
F(28.158807)	509.460						
Prob > F	0.000						
R2	0.082						
Adj R2	0.082

In terms of the explanatory variables, significant differences in TCL among age groups have been found for the entire study population 2006-2012 (Tables 1 and 2). Mean TCL steadily increased with age. Mean TCL in the 19-year-olds, the largest age group, was 4.151 mmol/l (95 % CI 4.146–4.156), with a total of 9.3 % having an elevated TCL (>5.17 mmol/l). Accordingly, TCL was lower in the younger age groups and higher in the older age groups (Table 1 and Fig. 2). Regarding socioeconomic occupational background (ISEI), young men from the 1st ISEI tertile “low” had the highest TCL (4.207 mmol/l, 95 % CI 4.200–4.212), whereas TCLs among the other occupational groups were in a similar range (4.155–4.169 mmol/l) and thus 0.038 to 0.045 mmol/l lower than those of the low-ISEI group (Table 1).

The positive association between TCL and BMI (categorized in accordance with the official WHO guidelines) is reported in Table 1. TCL steadily increased with BMI category. Whereas mean TCL was lower than 4.0 mmol/l among underweight young men (3.919 mmol/l, 95 % CI 3.902–3.936), mean TCL was ≥4.6 mmol/l in all WHO subcategories of obesity. Accordingly, only 4.0 % of the underweight young men had an elevated TCL (>5.17 mmol/l), compared with 27.5–32.9 % of the young men within the WHO subcategories of obesity. The regression coefficient for the BMI group 35.0–39.9 kg/m^2^ was the highest (0.682) compared with that of the normal weight men (BMI 18.5–24.9 kg/m^2^). However, in the most obese with a BMI ≥ 40.0 kg/m^2^, TCL started to decrease again, although not always to a significant degree.

There was significant variation in TCL when the study population was broken down according to the area of residence (*Grossregion*). The highest mean TCL was found in Région Lémanique (4.268 mmol/l, 95 % CI 4.259–4.277) and Zürich (4.243 mmol/l, 95 % CI 4.234–4.252), followed by Espace Mittelland (4.185 mmol/l, 95 % CI 4.178–4.192) and Ostschweiz (4.147 mmol/l, 95 % CI 4.139–4.155). Nordwestschweiz, Zentralschweiz, and Ticino all showed a mean TCL lower than 4.1 mmol/l (Tables 1 and 2).

Men performing well in the sports test showed a relatively low TCL. Consequently, young men with low sports performance levels (1st tertile) had the highest mean TCL, of 4.269 mmol/l (95 % CI 4.261–4.275), whereas the mean TCL in the group with the best sports test scores (3rd tertile) had a mean TCL of 4.089 mml/l (95 % CI 4.083–4.095). We repeated the multivariate regression with TCL as the dependent variable on both the full sample (including those conscripts without sports test results) and on the individual conscription years. The resulting coefficients (not shown) were very similar.

In a last step the TCLs were differentiated within the BMI categories according to the sports test performance tertiles (Table 3). Within the normal BMI and the overweight BMI groups, TCL decreased with increasing sports performance tertiles. For example, the differences in TCL between overweight young men with good sports test results (3rd tertile, mean TCL 4.443 mmol/l, 95 % CI 4.429–4.457) and young men with poor sports test results (1st tertile, mean TCL 4.29 mmol/l, 95 % CI 4.272–4.308) was 0.153 mmol/l (95 % CI 0.136–0.175, *p* < 0.001). However, in the underweight and the obese BMI groups mean TCL among sports performance tertiles did not differ markedly.

**Table 3:** TCL among male Swiss conscripts 2006-2012 adjusted for BMI and sports test performance (*Test Fitness Rekrutierung* TFR), *N* = 158,836

BMI	Sports Test	N	Mean	95 % CI	95 % CI	SD	N[5.17–6.18)	% [5.17–6.18)	N ≥ 6.18	% ≥ 6.18	N ≥ 5.17	% ≥ 5.17
<18.5	1st Tertile (low)	2121	3.907	3.879	3.935	0.665	67	3.2 %	9	0.4 %	76	3.6 %
	2nd Tertile (medium)	1813	3.924	3.893	3.955	0.681	68	3.8 %	9	0.5 %	77	4.3 %
	3rd Tertile (high)	923	3.927	3.887	3.967	0.620	34	3.7 %	2	0.2 %	36	3.9 %
	Total	4857	3.917	3.898	3.936	0.663	169	3.5 %	20	0.4 %	189	3.9 %
18.5–24.9	1st Tertile (low)	27907	4.113	4.104	4.122	0.743	2014	7.2 %	310	1.1 %	2324	8.3 %
	2nd Tertile (medium)	39827	4.077	4.070	4.084	0.707	2431	6.1 %	291	0.7 %	2722	6.8 %
	3rd Tertile (high)	47363	4.058	4.052	4.064	0.679	2675	5.6 %	261	0.6 %	2936	6.2 %
	Total	115097	4.078	4.074	4.082	0.705	7120	6.2 %	862	0.7 %	7982	6.9 %
25.0–29.9	1st Tertile (low)	13846	4.443	4.429	4.457	0.832	2169	15.7 %	402	2.9 %	2571	18.6 %
	2nd Tertile (medium)	10016	4.388	4.373	4.403	0.794	1326	13.2 %	239	2.4 %	1565	15.6 %
	3rd Tertile (high)	7223	4.290	4.272	4.308	0.765	740	10.2 %	130	1.8 %	870	12.0 %
	Total	31085	4.390	4.381	4.399	0.807	4235	13.6 %	771	2.5 %	5006	16.1 %
≥30.00	1st Tertile (low)	6394	4.688	4.666	4.710	0.887	1414	22.1 %	369	5.8 %	1783	27.9 %
	2nd Tertile (medium)	1144	4.624	4.573	4.675	0.874	266	23.3 %	42	3.7 %	308	27.0 %
	3rd Tertile (high)	259	4.677	4.575	4.779	0.837	54	20.8 %	11	4.2 %	65	25.0 %
	Total	7797	4.678	4.658	4.698	0.884	1734	22.2 %	422	5.4 %	2156	27.6 %
Total	1st Tertile (low)	50268	4.268	4.261	4.275	0.816	5664	11.3 %	1090	2.2 %	6754	13.5 %
	2nd Tertile (medium)	52800	4.143	4.137	4.149	0.742	4091	7.7 %	581	1.1 %	4672	8.8 %
	3rd Tertile (high)	55768	4.089	4.083	4.095	0.696	3503	6.3 %	404	0.7 %	3907	7.0 %
	Total	158836	4.164	4.160	4.168	0.755	13258	8.3 %	2075	1.3 %	15333	9.6 %

## Discussion

This study shows that the TCL of Swiss conscripts was significantly lower in 2011 and 2012 than in the conscription years 2006-2010: Mean TCL decreased from 4.225 mmol/l in 2006 to 4.100 mmol/l in 2012. A similar trend was found in the prevalence of conscripts with an elevated TCL ≥ 5.17 mmol/l (decrease from ≥10.2 % prior to 2011 to 6.9 % in 2011 and 8.2 % in 2012). Furthermore, a higher TCL was found to be associated with lower socioeconomic occupational status, lower sports test performance, higher BMI, higher age, and place of residence/*Grossregion* (higher TCLs in Région Lémanique and Zürich).

Previous studies of the TCL of Swiss young men conscripted in 2004, 2005, and 2004-2007 showed a similar prevalence of elevated TCL (9.1 %, 11 %, and 6.9 %, respectively) [14–16]. However, since these studies differ in terms of borderline high and high TCL limits and – even more importantly – in terms of applied laboratory test devices, reliable and direct comparison with the present study is not possible. Likewise, comparison with adult TCL in Switzerland is generally hindered by deviating study-population characteristics (sample composition, sample size, etc.) and measurement techniques. According to the 6th Swiss Nutrition Report, the prevalence of elevated cholesterol levels in the general adult Swiss population in 2007 was 18.1 % among men and 13.9 % among women [23]. In 1997, the prevalence had been lower (12.8 % in men and 10.9 % in women, respectively) [23].

Several studies focusing on cardiovascular and metabolic risks, including cholesterol levels, have drawn on datasets from other countries with mandatory military-conscription systems [11, 12, 24]. Mikkola et al. showed that among Finnish servicemen with an average age of 19 the prevalence of metabolic syndrome was comparable to that of adolescents in the USA and that a relatively high proportion of obese and overweight servicemen were diagnosed with metabolic syndrome [11]. A study of Norwegian recruits demonstrated that a high educational level of the father was associated with a lower BMI and better lipid profile [24]. Wallner et al. analyzed data of Austrian conscripts between 1986 and 2005 [12] and found that the mean TCL of these conscripts decreased over time from 4.318 mmol/l in 1986-1990 to 4.163 mmol/l in 2000-2005.

This study confirms that there are socioeconomic variations in Swiss conscripts’ TCL. Whereas Rühli et al. [14] found a relatively higher TCL among agricultural and construction workers conscripted in 2005, this study used the ISEI classification to analyze socioeconomic stratification. Again, conscripts with lower socioeconomic occupational background had the highest TCL. Panczak et al. found a similar relationship between low professional status and high BMI for the conscripts in 2004-2012 [17]. The strong positive relationship between TCL and BMI on the individual level was also confirmed by this study, which also offers a new finding: that there is no longer a marked increase in mean TCL among the three grades of obesity (BMI ≥ 30.0 kg/m^2^) as defined by the WHO. The positive association between TCL and age has also been shown for Swiss conscripts in earlier conscription years [14] and for the adult and elderly Swiss population in general [25]. Likewise, the relationship between better sports performance and lower TCL among the conscripts [14] is confirmed for the conscription years 2006-2012. This study adds that within the BMI categories of normal and overweight young men TCL was stratified by sports performance (higher performance = lower TCL). Consequently, TCL may help to differentiate between body mass as calculated according to the amount of body fat and lean body mass according to the amount of muscle mass. Including TCL into the analyses could thus help differentiating muscular from overweight young men in the BMI categories between 18.5–29.9 kg/m^2^ when no other information is available and were most of the BMI misjudgments regarding muscle or fat mass happen [17].

For the first time, regional differences in mean TCL among conscripts from the seven major regions of Switzerland were analyzed. In contrast to Rühli et al. [14] for conscripts in 2005, a high TCL was not restricted to young French-speaking men. In our study, the mean TCL of conscripts from the French-speaking Région Lémanique in 2006-2012 remained relatively high, but the TCL of conscripts from the Zürich area approached that of conscripts from Région Lémanique, and both were higher than the TCL of conscripts from the five other Swiss regions. This indicates that regional language backgrounds as an indicator of ethnocultural differences may no longer be the only determinant of variation in lipid profiles among young men. However, previous cross-sectional studies of cholesterol levels in the French-speaking Canton of Geneva and the city of Lausanne [26, 27] also indicate a higher prevalence of elevated cholesterol levels than that found among the Swiss population as a whole. What is needed next is an analysis of the TCL at a higher level of spatial resolution to determine whether urbanity or the neighborhood effect could account for these regional differences in TCL. In addition, there is a need for further analyses to determine precisely when and why TCL in Zürich started to catch up with the levels in Région Lémanique.

**Fig. 3:**
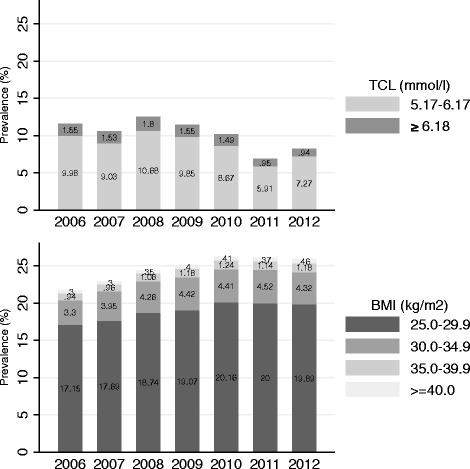
Prevalence trends of borderline high and high TCL (upper graph) and of overweight and obesity (lower graph) among Swiss conscripts 2006-2012 (*N* = 174,872)

**Fig. 4:**
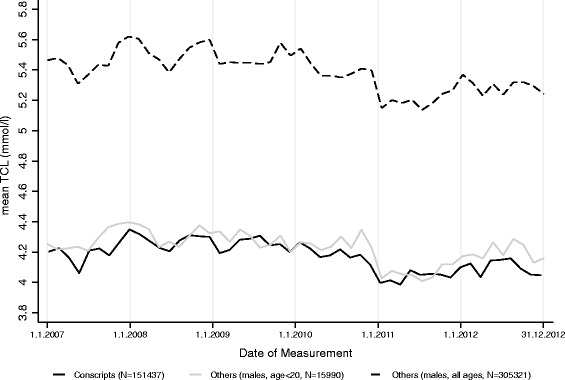
Daily mean TCL measurements (smoothed by local polynomial regressions, bandwidth 14) by the Viollier AG laboratory from 11/07/2006-31/12/2012 for all conscripts (*N* = 151,437)(solid black line), all other subjects (*N* = 305,321) (dashed line), and all other subjects aged below 20 years (*N* = 15,990) (solid grey line).

The significant decrease in Swiss conscripts’ overall TCL in 2011/2012 reported in this study may be explained by several possible factors: A) Studies of temporal variation in BMI among Swiss conscripts during the years 2004-2012 have shown that since 2010 the overweight and obesity epidemic seems to have stabilized [28]. Similarly, since 2006, after years of decline, the endurance performance of the conscripts in the sports test seems to have reached a plateau [19]. Together with reports of stabilizing weight (overweight and obese groups) among schoolchildren in Switzerland [29] these improvements suggest that the intensified public-health programs of the past five to ten years, focusing on nutrition and physical activity, are paying off [17]. Together with stabilizing BMI values among the examined conscripts the decrease in TCL in 2011/2012 may reflect this effort to improve lipid profiles and thus suggest that this may be one possible explanation (Fig. 3). B) In theory, TCL measurements could also be affected by possible biasing pre-analytic factors (time of measurements, feasting status, etc.) or small drifts in the laboratory analyses over the years, which cannot be controlled in this study. A comparison of all daily TCL measurements of the conscripts between 2006 and 2012 performed in the Viollier AG laboratory with the TCL measurements of all other male subjects (and as a sensitivity analysis also only with those aged below 20 years) suggests that the decrease towards the end of 2010 appears in all TCL measurements performed at the laboratory on the identical devices (Fig. 4). Thus, small drifts in the laboratory measurements may also contribute to the variation of annual mean TCL. However, the Swiss Armed Forces and the Viollier AG laboratory are rigorous in regard to the use of internal quality controls several times a day and periodic recalibration of the assay. C) Changes in the use of lipid-lowering medication over the years could partially explain changes in mean TCL. The only data available show that the percentage of Swiss men and women taking lipid-lowering medication steadily increased from 2002 to 2012 [30]. However, information on annual lipid-lowering drug usage in young men is not available. Overall, it is impossible to exclude one of these three possible explanations at this stage and further studies of TCL among Swiss conscripts are needed to determine whether or not TCL is indeed decreasing in the long-term.

The present study has several strengths. It relies on a large, representative sample and objectively measured data. The size of the sample permitted a comprehensive investigation of TCL and its association with several factors over a census cohort of 7 years. The mandatory conscription system of the Swiss Armed Forces meant that the sample was nationwide. The study has, however, some limitations. Cholesterol subfractions (LDL and HDL) would allow a more precise assessment of groups at higher disease risks than TCL alone. However, HDL and LDL were not available among the blood analyses by the Swiss Armed Forces. Also, more background information on the participants (smoking status, blood pressure, heart rate, etc.) would optimize the assessment of the risk profile. Unfortunately, such information is not recorded by the Swiss Armed Forces. However, from the Swiss Federal Survey of Adolescents we know that 29.9% of the young Swiss men smoked on a daily basis in 2010/2011 [31]. The analyses based on conscription data apply exclusively to young male Swiss nationals and may not represent lipid profiles of older age groups, women, and young men without Swiss citizenship – a group that is at particularly high risk of developing cardiovascular diseases later in life [32]. Another limitation is the classification of socioeconomic status by occupation at this young age level. About 8.5 % (N = 14,946) of the study population had not completed their schooling at the time of the conscription and therefore had yet to establish a place in the labor market [17, 33]. This issue was addressed by designating pupils as a separate occupational subgroup. In general, personal and medical data from conscription such as BMI, age, occupation, and place of residence are representative for more than 90 % of a given male birth cohort [17]. However, 65.8 % of the conscripts decided to participate in the voluntary blood-sample test in the analyzed 2006-2012 data. A possible participation bias was excluded by similar population characteristics of participants and non-participants, including age, BMI, socioeconomic background, and sports test performance (Appendix Table 4). The largest differences lay in the regional origin of the conscripts: Non-participants were more likely to be residents of Région Lémanique and Nordwestschweiz than of Zürich and Ostschweiz. All of the calculated percentages were surprisingly stable over time. Last but not least, BMI was the only body-shape measure available in the dataset. BMI is not an ideal measure of body composition since it does not precisely differentiate between weight associated with lean muscle mass and weight associated with fat mass [34]. However, because BMI is the most convenient measure available, it is the one most often used both in large-scale studies and in clinical practice [35]. Moreover, although waist circumference is theoretically a better measure for central obesity, waist circumference and body mass index are highly correlated and have the same ability to predict diabetes and cardiovascular disease [36, 37].

## Conclusion

In summary, the system of mandatory military conscription is a useful tool for disease risk monitoring in a given male population [12]. Our study helps to identify subgroups of young men with an unfavorable lipid profile who consequently are at risk of developing cardiovascular diseases later in life. In times of governmental budget cuts, reliable information about possible risk groups is crucial to the success of public health programs.

## Appendix

**Table 4:** Relative population characteristics of voluntarily blood sample participants (*N* = 174,872) and non-participants (*N* = 90,697) 2006-2012

	Year	2006^a^	2007	2008	2009	2010	2011	2012	Total
Overall	Participants	60.4 %	66.8 %	67.8 %	68.5 %	66.9 %	63.8 %	64.1 %	65.8 %
	Non-Participants	39.6 %	33.2 %	32.2 %	31.5 %	33.1 %	36.2 %	35.9 %	34.2 %
Age [18–19)	Participants	19.9 %	22.6 %	22.9 %	21.0 %	22.8 %	23.5 %	24.5 %	22.7 %
	Non-Participants	16.3 %	21.4 %	20.4 %	19.3 %	20.1 %	21.2 %	21.3 %	20.3 %
Age [19–20)	Participants	50.1 %	45.0 %	44.9 %	45.0 %	44.9 %	45.1 %	45.4 %	45.4 %
	Non-Participants	49.8 %	43.5 %	43.1 %	44.4 %	42.0 %	43.1 %	42.3 %	43.6 %
Age [20–21)	Participants	19.3 %	21.9 %	21.7 %	23.0 %	21.2 %	20.7 %	19.4 %	21.2 %
	Non-Participants	21.9 %	22.2 %	22.0 %	23.0 %	22.8 %	21.9 %	22.5 %	22.3 %
Overweight	Participants	17.7 %	17.8 %	19.3 %	19.9 %	20.6 %	20.5 %	20.4 %	19.6 %
	Non-Participants	16.6 %	17.8 %	18.0 %	17.7 %	19.7 %	19.4 %	19.4 %	18.6 %
Obese	Participants	4.3 %	4.5 %	5.1 %	5.5 %	5.5 %	5.6 %	5.6 %	5.2 %
	Non-Participants	4.9 %	6.7 %	6.8 %	7.0 %	7.1 %	6.8 %	6.7 %	6.7 %
ISEI low	Participants	30.6 %	31.0 %	31.0 %	30.3 %	30.5 %	30.1 %	28.8 %	30.3 %
	Non-Participants	29.1 %	31.0 %	30.2 %	30.2 %	29.6 %	29.0 %	28.0 %	29.6 %
ISEI high	Participants	25.7 %	24.6 %	25.5 %	26.1 %	25.4 %	24.3 %	26.0 %	25.3 %
	Non-Participants	26.6 %	24.3 %	27.9 %	28.2 %	27.1 %	26.3 %	28.4 %	27.0 %
Sport low	Participants	21.6 %	29.4 %	32.0 %	34.6 %	33.0 %	32.4 %	32.4 %	31.6 %
	Non-Participants	33.6 %	44.3 %	47.4 %	45.3 %	43.4 %	44.3 %	42.8 %	43.6 %
Sport high	Participants	48.6 %	36.4 %	33.5 %	32.8 %	33.6 %	34.0 %	35.0 %	35.1 %
	Non-Participants	33.0 %	22.7 %	22.5 %	23.2 %	24.8 %	24.1 %	25.6 %	24.7 %
Région Lémanique	Participants	17.9 %	14.9 %	15.4 %	16.1 %	14.1 %	14.6 %	15.8 %	15.3 %
	Non-Participants	14.6 %	14.7 %	21.1 %	19.6 %	18.1 %	17.2 %	20.1 %	18.1 %
Espace Mittelland	Participants	27.8 %	29.2 %	25.6 %	25.0 %	24.9 %	24.6 %	23.6 %	25.6 %
	Non-Participants	25.4 %	24.8 %	21.8 %	23.8 %	24.1 %	21.4 %	20.7 %	22.9 %
Nordwestschweiz	Participants	1.1 %	6.9 %	10.4 %	11.1 %	12.8 %	10.6 %	11.7 %	10.0 %
	Non-Participants	32.4 %	29.1 %	19.4 %	16.3 %	14.3 %	17.6 %	16.2 %	19.8 %
Zürich	Participants	17.0 %	15.6 %	15.8 %	14.6 %	15.8 %	17.5 %	16.3 %	16.0 %
	Non-Participants	6.9 %	8.2 %	9.7 %	11.5 %	14.2 %	15.5 %	14.2 %	11.9 %
Ostschweiz	Participants	19.8 %	18.5 %	19.7 %	18.6 %	17.9 %	18.8 %	18.0 %	18.6 %
	Non-Participants	7.9 %	10.3 %	10.7 %	13.0 %	14.0 %	13.0 %	13.4 %	12.1 %
Zentralschweiz	Participants	13.6 %	11.8 %	10.0 %	11.3 %	11.7 %	10.8 %	11.7 %	11.4 %
	Non-Participants	9.4 %	9.6 %	12.1 %	10.5 %	9.8 %	10.8 %	10.5 %	10.4 %
Ticino	Participants	2.8 %	3.0 %	3.1 %	3.3 %	2.8 %	3.1 %	2.9 %	3.0 %
	Non-Participants	3.5 %	3.4 %	5.2 %	5.4 %	5.6 %	4.6 %	5.0 %	4.7 %

^a^11/07/2006-31/12/2006
